# Supplementary material for: A Novel Synthetic Strategy for Preparing Polyamide 6 (PA6)-Based Polymer with Transesterification
Source: Polymers (Basel). 2019 Jun 3;11(6):978. doi: 10.3390/polym11060978 (PMC6631148; doi:10.3390/polym11060978)

Supplementary Materials:

Figure S1. DSC thermographs of cooling (**a**) different molecular weight prePA6. DSC thermographs of heating (**b**) different molecular weight prePA6.

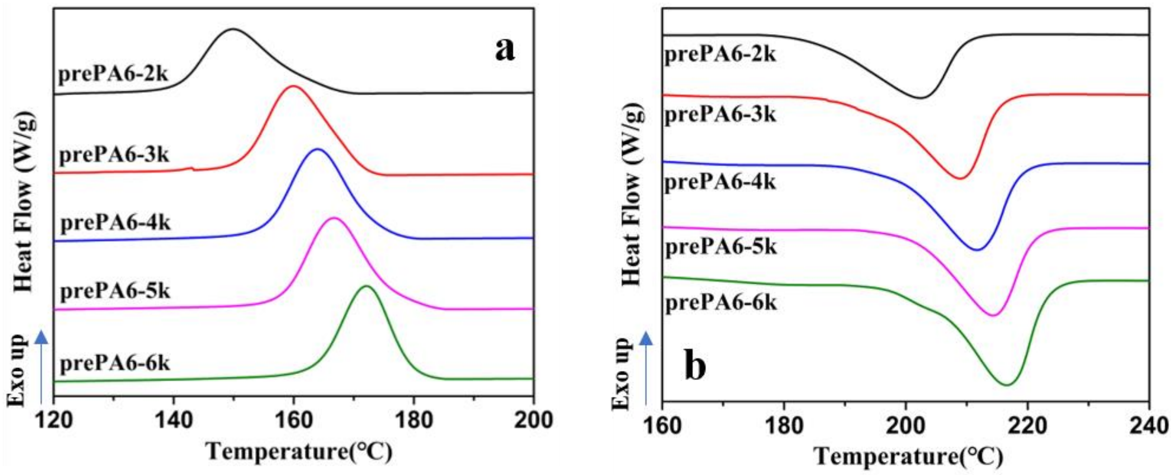

Figure S2. GPC of different samples.

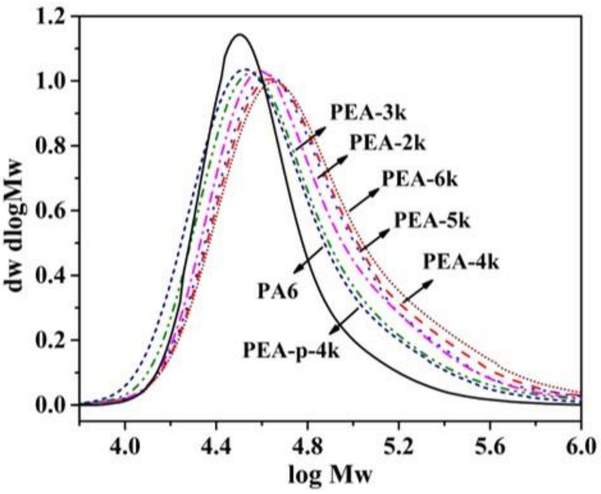

Supplement: Supplementary file 1 [file polymers-11-00978-s001.pdf]
